# ﻿Additions of New Endolichenic Fungi to Herpotrichiellaceae (Chaetothyriales, Ascomycota) from northern Thailand

**DOI:** 10.3897/mycokeys.120.153906

**Published:** 2025-07-30

**Authors:** Chanokned Senwanna, Jaturong Kumla, Pratthana Kodchasee, Nutchanan Duangkon, Nakarin Suwannarach

**Affiliations:** 1 Office of Research Administration, Chiang Mai University, Chiang Mai 50200, Thailand Chiang Mai University Chiang Mai Thailand; 2 Center of Excellence in Microbial Diversity and Sustainable Utilization, Chiang Mai University, Chiang Mai 50200, Thailand Chiang Mai University Chiang Mai Thailand; 3 Department of Biology, Faculty of Science, Chiang Mai University, Chiang Mai 50200, Thailand Chiang Mai University Chiang Mai Thailand

**Keywords:** Chaetothyriales, endolichenic fungi, fungal diversity, fungal taxonomy, lichen, new taxa

## Abstract

Endolichenic fungi associated with lichen thalli in Thailand are poorly known in terms of species diversity. During a study conducted in Chiang Mai Province, Thailand, in 2023, eight endolichenic fungal strains were isolated from healthy thalli of the foliose lichen, *Parmotrema* sp. These eight strains were identified as members of the family Herpotrichiellaceae using a combination of three nuclear ribosomal regions (ITS, LSU, and SSU), *tub2* sequence data, and morphological characteristics. Two strains of *Atrokylindriopsisracemosospora* and three strains of *Veronaeaendolichena* were identified as new species within the Herpotrichiellaceae, while three other strains were identified as the previously known species *Phialophorachinensis*. This study provides the first report of *P.chinensis* as an endolichenic fungal taxon and its first discovery in Thailand. Descriptions, illustrations, and phylogenetic placements of these eight strains are provided. Additionally, a discussion and update on the ecology and genera within the Herpotrichiellaceae are included. The findings of this study offer valuable information that enriches the diversity of endolichenic species associated with lichens in Thailand and contributes to enhancing our understanding of the ecology and taxonomy of the Herpotrichiellaceae.

## ﻿Introduction

The family Herpotrichiellaceae (Chaetothyriales, Eurotiomycetes) was established by Munk (1953) and typified by *Herpotrichiellamoravica*, which is synonymized under *Caproniapilosella* based on the teleomorph-anamorph connection (Müller et al. 1987; Untereiner 1997). The sexual morph of Herpotrichiellaceae is characterized by setose, ostiolate ascomata, bitunicate, saccate to ovoid asci with a thickened apex and long endotunica, and didymosporous, phragmosporous, or dictyosporous ascospores. The asexual morph consists of morphologically diverse dematiaceous hyphomycetes, predominantly characterized by black yeast (Müller et al. 1987; Untereiner et al. 1995; Arzanlou et al. 2007; Tian et al. 2021). These fungi primarily reproduce asexually, and exhibit limited morphological characters (Untereiner et al. 1995; Moreno et al. 2018; Tian et al. 2021). The internal transcribed spacer (ITS) and the large subunit (LSU) of ribosomal DNA sequences are commonly used to clarify the taxonomic placement of members in the Herpotrichiellaceae; however, many genera in this family have shown polyphyletic relationships (Quan et al. 2020; Tian et al. 2021; Torres-Garcia et al. 2023). Multi-gene sequence data are essential for resolving the morphological confusion among these genera and for establishing boundaries between genera and species. Species of Herpotrichiellaceae are not only recognized for causing human infections but also for their extremotolerance, thriving in a wide range of environments worldwide, including extreme conditions of high or low temperature, nutrient scarcity, desiccation, and solar irradiation (Müller et al. 1987; Untereiner 1997; Arzanlou et al. 2007; Teixeira et al. 2017; Muggia and Grube 2018; Quan et al. 2020, 2024; Chang et al. 2023; Thitla et al. 2023; Torres-Garcia et al. 2023; Diederich et al. 2024). Before this study, nineteen genera were recognized in this family, *viz. Aciculomyces*, *Aculeata*, *Atrokylindriopsis*, *Capronia*, *Cladophialophora*, *Exophiala*, *Fonsecaea*, *Marinophialophora*, *Melanoctona*, *Minimelanolocus*, *Petriomyces*, *Phialophora*, *Phaeoannellomyces*, *Pleomelogramma*, *Rhinocladiella*, *Thysanorea*, *Uncispora*, *Valentiella*, and *Veronaea* (Tian et al. 2021; Bezerra et al. 2022; Thitla et al. 2023; Torres-Garcia et al. 2023).

Lichens are ubiquitous on a wide range of surfaces and can occur in various environmental conditions (Müller 2001; Molnár and Farkas 2010; Lücking et al. 2017; Muggia and Grube 2018). They serve not only as bioindicators for assessing air pollution and evaluating environmental health, but also serve as sources of secondary metabolites with diverse pharmaceutical properties (Casale et al. 2015; Crawford 2019; Panta 2020; Wethalawe et al. 2021; Zhao et al. 2021). Lichen thalli provide a habitat conducive to the growth of other photosynthetic and non-photosynthetic organisms, such as algae, bacteria, filamentous fungi, and yeast, while shielding the photobiont from external harm (Suryanarayanan and Thirunavukkarasu 2017; Hawksworth and Grube 2020). Many lichen-associated fungi, including both endolichenic and lichenicolous fungi, are currently being studied and described at both morphological and molecular levels (Wang et al. 2016; Cometto et al. 2023, 2024; Si et al. 2023). Lichenicolous fungi are visible inhabitants of lichen thalli, whether they are host-specific parasites, saprotrophs, broad-spectrum pathogens, or commensals (Lawrey and Diederich 2003; Diederich et al. 2018). In contrast, endolichenic fungi are non-obligate fungi that colonize the internal tissues of lichen thalli without causing visible symptoms or negative effects. Their occurrence is similar to that of endophytic fungi (Arnold et al. 2009; Gao et al. 2016; Kellogg and Raja 2017; Elkhateeb and Daba 2021). Since lichenicolous fungi have been shown to be present in asymptomatic lichens, differentiating between lichenicolous and endolichenic fungi can be scientifically challenging (Yang et al. 2022). Species diversity of endolichenic fungi influenced not only by lichen host species but also by the environment, habitat, host, and geographic distribution (U’Ren et al. 2012; Gao et al. 2016; Muggia and Grube 2018; Agrawal et al. 2020; Elkhateeb and Daba 2021). Moreover, studies on the diversity of endolichenic fungi in recent years have relied on culture-based and metabarcoding approaches (U’Ren et al. 2010; Zhang et al. 2016; Fernández-Mendoza et al. 2017; Yang et al. 2021; Si et al. 2023). According to several previous studies, ascomycetes are the dominant group of endolichenic fungi associated with lichens, followed by basidiomycetes and mucoromycetes. (Gao et al. 2016; Muggia and Grube 2018; Chakarwarti et al. 2020; Elkhateeb and Daba 2021; Cometto et al. 2024). Concurrently, various endolichenic fungal genera associated with lichen thalli worldwide have been identified, including *Aspergillus*, *Chaetomium*, *Cladophialophora*, *Cladosporium*, *Penicillium*, *Rhinocladiella*, *Trichoderma*, and *Xylaria* (Muggia et al. 2017; Maduranga et al. 2018; Muggia and Grube 2018; Yang et al. 2021; Cometto et al. 2023, 2024). Thailand has a humid tropical climate and is recognized as one of the most biodiverse countries in the world (Tungmunnithum et al. 2022; Kaewsangsai et al. 2024). Numerous studies have been conducted on bacteria, insects, plants, fungi (endophytes, saprophytes, and pathogens), and lichens; however, the diversity of endolichenic fungi remains relatively unexplored (Tanasupawat 2009; Maneechan and Prommi 2015; Dathong 2016; Buaruang et al. 2017; Hyde et al. 2018; Jayawardena et al. 2020; Tayung 2022; Bamrungpanichtavorn et al. 2023; Krongdang et al. 2023; Kaewsangsai et al. 2024; Nimnoi et al. 2024). Therefore, this research aimed to study the diversity of endolichenic fungi in Thailand. During a 2023 study of endolichenic fungi in northern Thailand, eight fungal strains belonging to the family Herpotrichiellaceae were isolated from foliose lichen thalli (*Parmotrema* sp.), including three strains of *Phialophorachinensis* and five strains of unidentified fungal taxa. Based on morphology, growth temperature, and multi-gene phylogenetic analyses, five Herpotrichiellaceous fungi are identified as novel taxa. Additionally, we provide an update on the ecology and genera within the Herpotrichiellaceae family.

## ﻿Materials and methods

### ﻿Lichen collection

The foliose lichen thalli of *Parmotrema* sp. were collected from Chai Prakan and Mueang Chiang Mai Districts, Chiang Mai Province, northern Thailand, during June to July 2023. During the collection period, Chai Prakan District experienced daily rainfall of 81.2 mm, with a temperature range of 27 °C to 36 °C, whereas Mueang Chiang Mai District received daily rainfall of 153.2 mm, with temperatures ranging from 24 °C to 34 °C. Healthy specimens were carefully removed from tree bark using a sterile knife, transferred into individual plastic bags, the collection details were recorded (Rathnayaka et al. 2024), and the specimens were stored at 4 °C until processing within 48–96 hours after sampling.

### ﻿Fungal isolation

The lichen thalli were carefully observed under an Olympus SZ40 stereo microscope to avoid damage and prevent contamination of any parts. Fungal isolation was performed following the protocols of Maduranga et al. (2018) and Si et al. (2023), with some modifications. The healthy lichen thalli were thoroughly washed in running water for 10 min to remove excess dirt and dried with sterile paper towels. The thalli were randomly dissected aseptically into 1 cm^2^ fragments using a sterile razor blade under a stereo microscope, followed by surface sterilization with immersion in 70% ethanol for 1 min, 1% NaOCl for 1 min, and rinsing three times in sterile distilled water for 1 min. The upper and lower cortex layers of surface-sterilized lichen fragments were carefully scratched under a stereo microscope using a sterile surgical blade and tweezers. The medulla tissue was carefully removed and cleaned with sterile distilled water. The fragment was then cut into a 0.5 cm^2^ segment. Six lichen segments were evenly placed in a 9 cm Petri dish containing potato dextrose agar (PDA; Condalab, Laboratorios Conda S.A., Spain) and dichloran rose-bengal agar (DRBC; Difco, Becton, Dickinson and Company, USA). For each specimen, three replications of the isolation plates were made. The plates were incubated at 25 °C until the growth of endolichenic fungi. The fungal hypha that grew from the segments was transferred onto freshly prepared PDA plates and incubated at 25 °C. The pure fungal strains were stored short-term on PDA slants at 4 °C and long-term in 20% glycerol at –80 °C at the Sustainable Development of Biological Resources culture collection (SDBR-CMU), Faculty of Science, Chiang Mai University, located in Chiang Mai Province, Thailand. Additionally, fungal strains were deposited and permanently maintained in a metabolically inactive state at the Chiang Mai University Biology Department’s Herbarium (CMUB), Chiang Mai University, Chiang Mai Province, Thailand. New fungal taxa were registered in the MycoBank database (MycoBank 2025).

### ﻿Morphological studies

Pure fungal colonies were investigated on different media for induce pigmentation and sporulation viz., corn meal agar (CMA; Difco, BBL™, USA), cornmeal dextrose agar (CMD; Difco corn meal agar + 2% dextrose), malt extract agar (MEA; Gibco, Life Technologies Corporation, USA), oatmeal agar (OA; Difco, Becton, Dickinson and Company, USA) and potato carrot agar (PCA; 200 g of each boiled and filtered carrots and potatoes, 17 g agar, 1 L distilled water). Mycelial plugs (3 mm × 3 mm) from 7-day-old PDA culture were cultured on each medium. The plates were incubated at 25 °C. In addition, the cardinal growth temperatures at 4, 25, 30, and 35 °C of fungal strains were determined on MEA for two weeks in the dark. Microscopic features, including mycelia, branching patterns, and sporulating structures, were observed and photographed using a Nikon DS-Ri2 camera connected to a Nikon ECLIPSE Ni (Tokyo, Japan) compound microscope. The fungal structure measurement was performed using the Tarosoft Image Framework program (v. 0.9.0.7). Adobe Photoshop version 22.4.2 (Adobe Systems U.S.A.) was employed to create the photographic plates.

### ﻿DNA extractions, polymerase chain reaction, and sequencing

DNA extractions were performed using the Fungal DNA Extraction Kit (FAVORGEN, Ping-Tung, Taiwan) from mycelium scraped from colonies grown on PDA using a sterile scalpel. DNA concentration and quality were determined by NanoDrop One^C^ Microvolume UV-Vis Spectrophotometer (Thermo Scientific, Wilmington, DE, USA). The nuclear ribosomal internal transcribed spacer (ITS) region, 28S large subunit (LSU), 18S small subunit (SSU), and beta tubulin gene (*tub2*) were amplified using ITS4/ITS5 (White et al. 1990), LR0R/LR5 (Vilgalys and Hester 1990), NS1/NS4 (White et al. 1990), and Bt2a/Bt2b (Glass and Donaldson 1995) primers, respectively. Polymerase chain reaction (PCR) amplification was performed using peqSTAR thermal cycler (PEQLAB Ltd., Fareham, UK) in a final volume of 20 μL containing 10 µL of 2 × Quick TaqTM HS DyeMix (TOYOBO, Japan), 6 µL of sterile deionized water, 1 µL of 10 µM of each forward and reverse primer, and 2 µL of DNA. The PCR conditions for ITS, LSU, and SSU amplification were as follows: initial denaturing step of 95 °C for 5 min, followed by 35 cycles of denaturation at 94 °C for 30 s, annealing at 52 °C for 45 s, elongation at 72 °C for 1 min, and final extension at 72 °C for 10 min. The PCR conditions for *tub2* amplification were as follows: initial denaturing step of 94 °C for 4 min, followed by 35 cycles of denaturation at 94 °C for 40 s, annealing at 52 °C for 30 s, elongation at 72 °C for 1 min, and final extension at 72 °C for 7 min. The PCR product was checked by 1% agarose electrophoresis gels under UV light. PCR clean-up Gel extraction NucleoSpin® Gel and PCR Clean-up Kit (Macherey-Nagel, Germany) was used to purify the PCR products according to the manufacturer’s protocol, which were sequenced by 1^st^ BASE Company (Kembangan, Malaysia). New sequences generated in this study were deposited in GenBank.

### ﻿Sequence alignment and phylogenetic analyses

The ITS, LSU, SSU, and *tub2* sequence data were edited, quality-checked, and assembled using the SeqMan 5.00 software. The consensus sequences were BLAST-searched using the NCBI nucleotide database (http://blast.ncbi.nlm.nih.gov/) to assess the closely related species. Sequences generated in the analyses were chosen from related sequences of the genera in Herpotrichiellaceae which were derived from GenBank and recent publications (Su et al. 2023; Thitla et al. 2023; Torres-Garcia et al. 2023). *Cyphellophoralaciniata* (CBS 190.61) and *C.suttonii* (CBS 449.91) were selected as the out-group taxa (Table 1).

**Table 1.** Taxa used in this study, along with their corresponding GenBank accession numbers. Ex-type strains are indicated with superscript “T”. The taxa obtained in this study are in bold. “–” is indicated the absence of sequence data in GenBank.

Taxa name	Strain number	GenBank Accession number			
		ITS	LSU	SSU	*tub2*
*Aciculomycesrestrictus*	FMR 18994^T^	ON009870	ON009950	–	ON667802
*Aculeataaquatica*	MFLUCC 11-0529 ^T^	MG922571	MG922575	MG922579	–
*Atrokylindriopsissetulosa*	HMAS245592 ^T^	KP337330	KP337329	–	–
*Atrokylindriopsisracemosospora*	SDBR-CMU502 ^T^	PQ525388	PQ525396	PQ525404	PQ523731
*Atrokylindriopsisracemosospora*	SDBR-CMU503	PQ525389	PQ525397	PQ525405	–
*Caproniaacutiseta*	CBS 618.96 ^T^	AF050241	KF155191	AJ232942	–
*Caproniacamelliae-yunnanensis*	CGMCC 3.19061 ^T^	MH807377	MH807378	MH807379	–
*Caproniadactylotricha*	CBS 604.96 ^T^	AF050243	KX712343	AJ232943	–
*Capronialeucadendri*	CBS 122672 ^T^	EU552108	MH874754	–	–
*Capronianigerrima*	CBS 513.69	MH859363	AY605075	AY541478	–
*Caproniapilosella*	AFTOL-ID 657 ^T^	DQ826737	DQ823099	DQ823106	–
*Cladophialophoraabundans*	CBS 126736 ^T^	KC776592	KC812100	–	–
*Cladophialophoraarxii*	CBS 306.94 ^T^	EU103986	KX822320	–	–
*Cladophialophorabantiana*	CBS 173.52 ^T^	EU103989	KF155189	–	–
*Cladophialophoracarrionii*	CBS 160.54 ^T^	EU137266	FJ358234	FJ358302	EU137201
*Cladophialophoradenticulata*	FMR 18992 ^T^	ON009845	ON009925	–	ON667783
*Cladophialophoradevriesii*	CBS 147.84 ^T^	EU103985	KC809989	AJ232947	–
*Cladophialophoraemmonsii*	CBS 979.96 ^T^	EU103996	–	–	–
*Cladophialophoraexuberans*	CMRP1227 ^T^	KY680429	KY570931	–	KY689826
*Cladophialophoraheterospora*	FMR 18641 ^T^	ON009847	ON009927	–	ON491592
*Cladophialophoraimmunda*	CBS 834.96 ^T^	MH862619	KC809990	KF155194	EU137203
*Cladophialophorainabaensis*	EUCL1 ^T^	LC128795	–	–	–
*Cladophialophoralanosa*	KNU 16032 ^T^	LC387460	LC387461	–	–
*Cladophialophoramycetomatis*	CBS 122637 ^T^	FJ385276	KX822321	KX822278	–
*Cladophialophoranyingchiensis*	CGMCC 3.17330 ^T^	MG012699	MG197824	MG012728	MG012747
*Cladophialophorapsammophila*	CBS 110553 ^T^	AY857517	KX712346	–	–
*Cladophialophorarecurvata*	CBS 143843 ^T^	LT985878	LT985879	–	LT985894
*Cladophialophorarupestricola*	SDBR-CMU446 ^T^	OP903465	OP903502	OR141860	OR139230
*Cladophialophorasamoensis*	CBS 259.83 ^T^	MH861581	KC809992	KX822281	EU137174
*Cladophialophorasribuabanensis*	SDBR-CMU476 ^T^	OQ991178	OQ979608	OR141868	OR139238
*Cladophialophorasubtilis*	CBS 122642 ^T^	FJ385273	KX822322	KX822283	–
*Cladophialophoratengchongensis*	CGMCC3.15201 ^T^	MG012702	MG197827	MG012731	MG012750
*Cladophialophorathailandensis*	SDBR-CMU451 ^T^	OP903470	OP903507	OR141865	OR139235
*Cladophialophoratumulicola*	JCM 28766 ^T^	LC192098	LC192063	–	–
*Cladophialophorayegresii*	CBS 114405 ^T^	EU137322	KC809994	KX822284	EU137209
*Cyphellophoralaciniata*	CBS 190.61 ^T^	EU035416	FJ358239	FJ358307	JQ766329
*Cyphellophorasuttonii*	CBS 449.91 ^T^	KC455243	KC455256	KC455300	KC455226
*Exophialabonariae*	CBS 139957 ^T^	JX681046	KR781083	–	–
*Exophialabrunnea*	CBS 587.66 ^T^	MH858890	KX712342	JN856013	JN112442
*Exophialacandelabrata*	FMR 18336 ^T^	ON009851	ON009931	–	ON491591
*Exophialaopportunisticica*	CBS 109811 ^T^	KF928437	KF928501	–	JN112486
*Exophialapalmae*	UPCB 86822 ^T^	KY680434	KY570929	–	KY689829
*Exophialapisciphila*	CBS 537.73 ^T^	DQ826739	AF050272	DQ823108	JN112493
*Exophialaradicis*	P2854 ^T^	KT099204	KT723448	KT723453	KT723463
*Exophialasalmonis*	CBS 157.67 ^T^	JF747137	AY213702	EF413608	JN112499
*Exophialasiamensis*	SDBR-CMU417 ^T^	ON555811	–	ON555826	ON544240
*Exophialasiamensis*	SDBR-CMU418	ON555812	–	ON555827	ON544241
*Fonsecaeabrasilliensis*	CBS 119710 ^T^	JN173784	KF155183	KF155203	JN368478
*Fonsecaeaerecta*	CBS 125763 ^T^	KC886414	KF155186	KF155210	KF155221
*Fonsecaeamonophora*	CBS 102243 ^T^	EU938579	FJ358247	FJ358315	EU938542
*Fonsecaeamultimorphosa*	CBS 980.96 ^T^	JF267657	KF155188	JF433950	HQ681121
*Fonsecaeapedrosoi*	CBS 271.37 ^T^	AB114127	KJ930166	AY554290	EU938559
*Marinophialophoragarethjonesii*	MFLUCC 16-1449	KY305174	KY305177	KY305178	–
*Marinophialophoragarethjonesii*	KUMCC 16-0066 ^T^	KY305175	KY305176	KY305179	–
*Melanoctonatectonae*	MFLUCC 12-0389 ^T^	KX258778	KX258779	KX258780	–
*Petriomycesobovoidisporus*	SDBR-CMU478^T^	OQ991180	OQ979610	OR141870	OR139240
*Petriomycesobovoidisporus*	SDBR-CMU479	OQ991181	OQ979611	OR141871	OR139241
*Phaeoannellomyceselegans*	CBS 122.95 ^T^	KY115190	–	–	–
*Phaeoannellomyceselegans*	CBS 101597	KF928443	KF928507	–	KF928571
*Phialophoraamericana*	CBS 281.35	EU514694	EU514694	–	EU514707
*Phialophoraamericana*	CBS 400.67	EU514695	EU514695	–	EU514708
*Phialophoraamericana*	UAMH 10875 ^T^	EU514696	EU514696	–	EU514712
*Phialophoraamericana*	UAMH 10876	EU514697	EU514697	–	EU514713
*Phialophorachinensis*	BMU 02669	KF881930	KJ930045	–	KF971731
*Phialophorachinensis*	CBS 140326 ^T^	KF881964	KJ930093	KM658060	KF971765
*Phialophorachinensis*	IFM 51934	AB550779	AB550779	–	–
*Phialophorachinensis*	BMU 00447	KF881957	KJ930082	–	KF971757
*Phialophorachinensis*	BMU 00150	KJ700947	KJ930077	–	KM658123
*Phialophorachinensis*	BMU 01057	KJ700953	KJ930086	–	KM658129
*Phialophorachinensis*	BMU 07661	KJ701013	KJ930155	–	KM658101
*Phialophorachinensis*	BMU 07656	KJ700998	KJ930152	–	KM658107
*Phialophorachinensis*	BMU 07664	KJ701003	KJ930156	–	KM658111
*Phialophorachinensis*	SDBR-CMU504	PQ525390	PQ525398	PQ525406	PQ523732
*Phialophorachinensis*	SDBR-CMU505	PQ525391	PQ525399	PQ525407	PQ523733
*Phialophorachinensis*	SDBR-CMU506	PQ525392	PQ525400	PQ525408	PQ523734
*Phialophoraellipsoidea*	CBS 286.47 ^T^	AF050282	AF050282	–	EU514715
*Phialophoraellipsoidea*	CBS 224.97	U31848	–	–	KU306354
*Phialophoraexpanda*	BMU 01245	KF881934	KJ930088	–	KF971734
*Phialophoraexpanda*	CBS 140298 ^T^	KF881937	KJ930095	–	KF971737
*Phialophoramacrospora*	CBS 273.37 ^T^	AF050281	AF050281	–	EU514714
*Phialophoramacrospora*	CBS 138.67	KU306356	–	–	KU306355
*Phialophoratarda*	CBS 111589 ^T^	KU306362	–	–	KU306347
*Phialophorasubmersa*	FMR 17150 ^T^	ON009864	ON009944	–	ON667800
*Phialophorasubmersa*	FMR 18996	ON009867	ON009947	–	ON667798
*Phialophorasubmersa*	FMR 18997	ON009868	ON009948	–	ON667799
*Phialophoraverrucosa*	CBS 140325 ^T^	NR146242	KJ930073	NG061187	KF971761
*Rhinocladiellaanceps*	CBS 181.65 ^T^	EU041805	EU041862	AY554292	–
*Rhinocladiellaatrovirens*	CBS 317.33 ^T^	AB091215	MH866906	–	–
*Rhinocladiellamackenziei*	CBS 650.93 ^T^	AY857540	AF050288	–	–
*Rhinocladiellaphaeophora*	CBS 496.78 ^T^	EU041811	EU041868	EF137366	GU079661
*Rhinocladiellaquercus*	CBS 141448 ^T^	KX306769	KX306794	–	–
*Thysanoreaaquaticus*	MFLUCC 15-0414	KR215607	KR215612	KR215617	–
*Thysanoreaclavatus*	DLU 3022 ^T^	MT271774	MT271772	MT271777	–
*Thysanoreapapuana*	CBS 212.96 ^T^	MH862572	MH874198	–	–
*Thysanoreasubmersus*	KUMCC 15-0206^T^	KX789212	KX789215	–	–
*Thysanoreathailandensis*	MFLUCC 15-0971^T^	MG922573	MG922577	MG922581	–
*Uncisporasinensis*	YMF 1.03683 ^T^	KU173860	KU558914	KU558913	–
*Uncisporawuzhishanensis*	YMF 1.04080 ^T^	KU173859	KU558912	KU558911	–
*Valentiellamaceioensis*	BSS 376	MZ042488	MZ042486	–	–
*Valentiellamaceioensis*	CBS 141892 ^T^	KY305141	KX348014	–	–
*Veronaeaaquatica*	JAUCC2549 ^T^	MW046892	MW046893	–	MW248394
*Veronaeabotryosa*	CBS 254.57 ^T^	MH857711	MH869255	JN856021	JN112505
*Veronaeabotryosa*	MFLUCC 11-0072	MG922570	MG922574	MG922578	–
*Veronaeabotryosa*	CBS 102593	KF928429	KF928493	–	KF928557
*Veronaeabotryosa*	CBS 572.90	MH862237	MH873920	–	–
*Veronaeabotryosa*	GZCC 19-0557	OP377853	OP377938	OP378020	–
*Veronaeacompacta*	CBS 268.75 ^T^	EU041819	EU041876	–	–
*Veronaeaendolichena*	SDBR-CMU507^T^	PQ525393	PQ525401	PQ525409	PQ523735
*Veronaeaendolichena*	SDBR-CMU508	PQ525394	PQ525402	PQ525410	PQ523736
*Veronaeaendolichena*	SDBR-CMU509	PQ525395	PQ525403	PQ525411	PQ523737
*Veronaeajaponica*	CBS 776.83 ^T^	EU041818	EU041875	–	–
*Veronaeapolyconidia*	CGMCC 3.25589 ^T^	OR807862	OR807868	OR807865	OR817660
*Veronaeapolyconidia*	UESTCC 23.0138	OR807863	OR807869	OR807866	OR817661
*Veronaeapolyconidia*	UESTCC 23.0139	OR807861	OR807867	OR807864	OR817659

The alignment of each locus was conducted using the MAFFT sequence alignment server (Katoh et al. 2019; http://mafft.cbrc.jp/alignment/server/) and improved manually where necessary in BioEdit v.7.0.9.1 (Hall 1999). Sequence data for ITS and LSU were analyzed individually to examine the incongruence in the topology of the phylogenetic tree. Concatenated ITS, LSU, SSU, and *tub2* sequence data were then analyzed and used to generate phylogenetic trees based on maximum likelihood (ML) and Bayesian inference (BI) methods.

The ML tree was executed using the GTRGAMMA substitution model of nucleotide substitution and set to 1,000 bootstrapping replicates via the CIPRES web portal. The BI tree was executed using a Markov Chain Monte Carlo (MCMC) algorithm with MrBayes v. 3.2.6 (Ronquist et al. 2012), employing the best-fit model of sequence evolution determined by MrModeltest v. 2.3 (Nylander 2008). The GTR+I+G substitution model was the best-fitting model of sequence evolution, which was determined based on the Akaike Information Criterion (AIC) using the MrModeltest v2.3 (Nylander 2008) implemented in PAUP v. 4.0b10 (Swofford 2002). Parameters for Bayesian inference: Four simultaneous Markov chains were set to run 20,000,000 generations, with the tree sampling every 100^th^ generation, resulting in 200,000 trees. The first 25% of generated trees as part of a burn-in procedure were discarded, and the remaining trees were evaluated for posterior probabilities (PP) of a majority rule consensus tree. The resulting trees from ML and BI were displayed using FigTree v1.4.0 (Rambaut 2016) and modified using Adobe Illustrator Version 25.2.3 and Adobe Photoshop Version 22.4.2 (Adobe Systems, United States of America).

## ﻿Results

### ﻿Phylogenetic analyses

**Figure 1.**
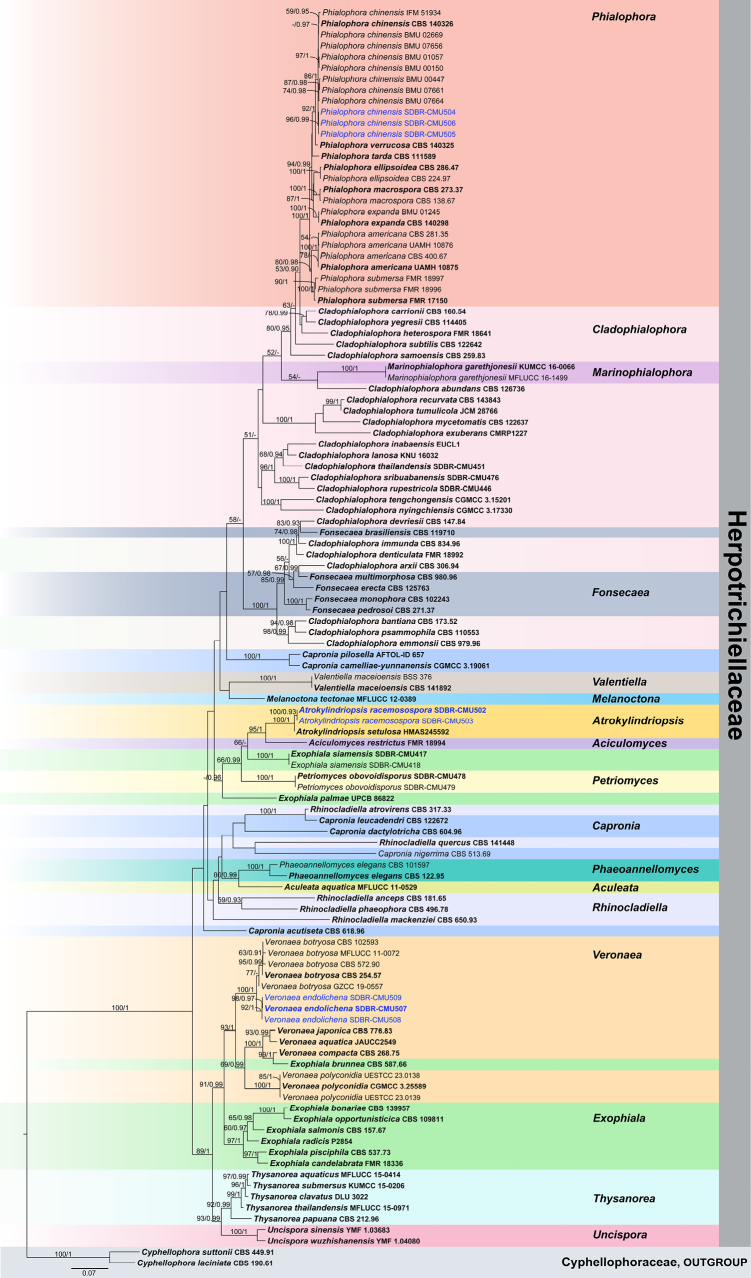
Phylogram generated from maximum likelihood analysis of members in Herpotrichiellaceae based on a combined ITS, LSU, SSU, and *tub2* sequence dataset. Bootstrap values ≥ 50% MLBS and Bayesian posterior probabilities (PP) ≥ 0.90 are shown above nodes and defined as ML/PP. The tree is rooted to *Cyphellophoralaciniata* (CBS 190.61) and *C.suttonii* (CBS 449.91). The fungal strains obtained in this study are blue. Ex-type strains are in bold. The scale bar represents the expected number of nucleotide substitutions per site.

The concatenated ITS, LSU, SSU, and *tub2* sequence dataset consists of 114 representative strains of species in the families Herpotrichiellaceae and Cyphellophoraceae (outgroup). The total alignment length comprises 3,413 characters (ITS: 1–779; LSU: 780–1,644; SSU: 1,645–2,872; *tub2*: 2,873–3,413), including gaps. The topologies of the trees generated from ML and BI analyses were congruent. The resultant ML tree is shown in Fig. 1. The best RAxML tree with a final likelihood optimization value of -32004.987525 is presented. The matrix contained 1,459 distinct alignment patterns, with 38.84% of the characters being undetermined or gaps. Estimated base frequencies were as follows: A = 0.251383, C = 0.234823, G = 0.265952, T = 0.247842; substitution rates AC = 1.510868, AG = 3.198001, AT = 1.566806, CG = 0.906099, CT = 6.013302, GT = 1.000000; gamma distribution shape parameter α = 0.548282. The average standard deviation of split frequencies was 0.009767 at the end of total MCMC generations.

The phylogenetic tree, based on the analysis of a combined ITS, LSU, SSU, and *tub2* sequence data, shows the relationships of taxa within the family Herpotrichiellaceae (Fig. 1). The results indicated that all eight endolichenic fungal strains obtained in this study belong to the family Herpotrichiellaceae. Three strains (SDBR-CMU504, SDBR-CMU505, and SDBR-CMU506) clustered within the *Phialophora* species and were found to be phylogenetically related to *P.chinensis*, with 86% ML and 1 PP statistical support. Interestingly, the other five strains formed distinct lineages from previously known taxa. Two fungal strains (SDBR-CMU502 and SDBR-CMU503) of a novel taxon formed a monophyletic clade clustering with *Atrokylindriopsissetulosa*, with 100% ML and 1 PP support values. In addition, three remaining strains (SDBR-CMU507, SDBR-CMU508, and SDBR-CMU509) are also introduced as a new species, which forms a distinct lineage in *Veronaea* with 92% ML and 1 PP and is in a sister clade to *V.botryosa*.

### ﻿Taxonomic descriptions

#### ﻿ Atrokylindriopsis racemosospora

Taxon classificationFungiChaetothyrialesHerpotrichiellaceae

Senwanna, J. Kumla & N. Suwannar. sp. nov.

CA403195-9F59-5314-80C9-2B3B9C33CDF8

858499

Fig. 2

##### Etymology.

In reference to the spore arrangement resembling a raceme form.

##### Type.

Thailand • Chiang Mai Province: Chai Prakan District, Nong Bua Subdistrict, endolichenic from the medulla of foliose lichen (*Parmotrema* sp.) on *Prunusdomestica*, 19°42'23"N, 99°1'32"E, elevation 1160 m, 2 June 2023, C. Senwanna and N. Suwannarach, CMUB40067 (***Holotype***, preserved in a metabolically inactive state. ***Ex-type*** living culture LC05-1 = SDBR-CMU502).

**Figure 2.**
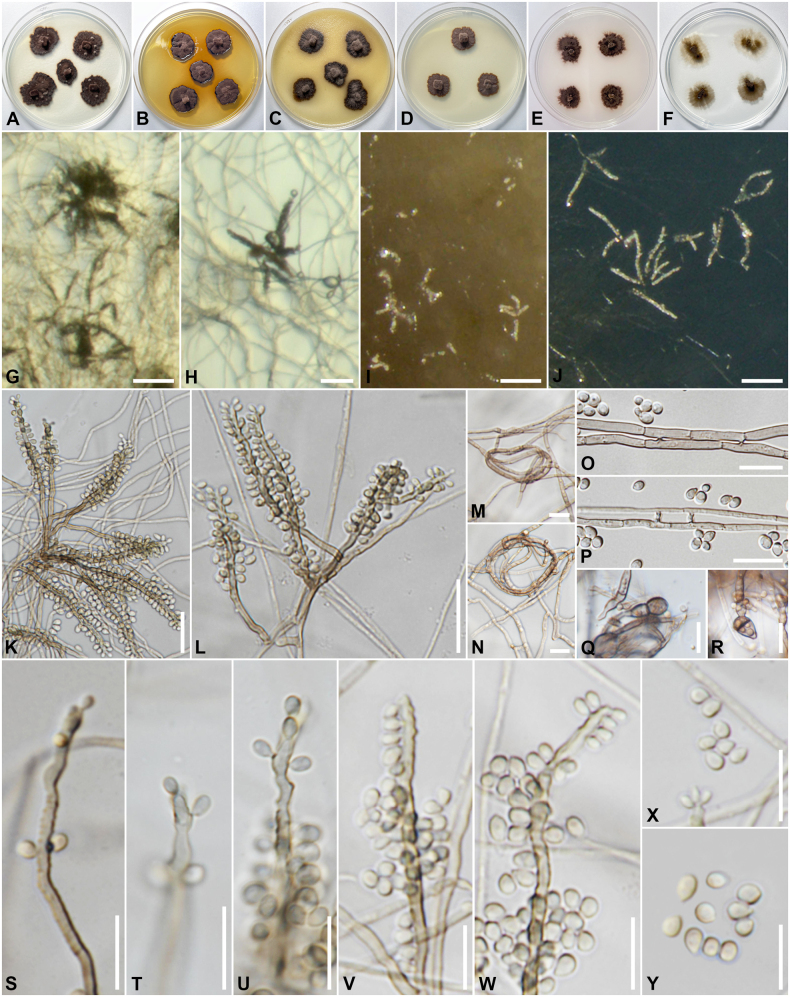
*Atrokylindriopsisracemosospora* (SDBR-CMU502, ex-type). A–F. Colonies on PDA, MEA, OA, PCA, CMD and CMA, respectively, after 48 d at 25 °C; G, H. Conidiophores sporulating on PCA; I. Conidiophores sporulating on OA; J. Conidiophores sporulating on CMA; K, L. Conidiophores and conidia; M–N. Hyphal coil; O, P. Hyphal anastomosis; Q, R. Terminal chlamydospores; S–W. Conidiophores with conidiogenous loci; X–Y. conidia. Scale bars: 10 µm (G–J); 20 µm (K–L); 10 µm (M–Y).

##### Cultural characteristics.

Colonies on different agar media were incubated in the dark at 25 °C for 2 months; colonies flat, irregular, with edge undulate, velvety; on PDA (18 to 27 mm in diameter) surface grayish brown, light brown at the margin, reverse olivaceous black, light brown at the margin; on MEA (18 to 23 mm in diameter) surface grayish brown, dark brown to black at the margin, reverse olivaceous black, producing brown pigment in agar; on OA (17 to 23 mm in diameter) surface grayish brown, reverse olivaceous black, sporulation absent; on PCA (17 to 20 mm in diameter) surface and reverse brownish gray, reverse olivaceous black, sporulation absent. On CMD (15 to 20 mm in diameter) surface and reverse grayish brown; and on CMA (20 to 27 mm in diameter) surface and reverse brownish grey, sporulation absent. ***Asexual morph in vitro*** dematiaceous hyphomycetes. ***Hyphae*** 1–2.5 µm wide, pale brown, simple to branched, septate, smooth, thin-walled, coiling, anastomosis observed. ***Conidiophores*** (10–)20–111(–153) × (1.4–)1.8–2.6(–2.9) µm (*x̄* = 53.17 × 2.15, *n* = 40), macronematous, monomematous, straight or slightly flexuous, unbranched, continuous or 1–3 septate, dark brown, paler terminally, smooth-, thick-walled. ***Conidiogenous cells*** (6.6–)15.5–36(–45.5) × (1.4–)1.8–2.6(–2.9) µm (*x̄* = 25.5 × 2.16, *n* = 40), integrated, polyblastic, terminal to mostly intercalary, proliferating sympodial and producing conidia from short denticles, subcylindrical, pale brown to brown, fertile parts subhyaline; denticles scattered, slightly darkened, 0.4–1 µm wide. ***Conidia*** (2.5–)2.8–4.4(–5) × (1.8–)2–3.3 µm (*x̄* = 3.72 × 2.68, *n* = 40), abundant, obovoid or subglobose, with a round apex, and slightly truncate base, aseptate, subhyaline to pale brown, smooth-walled, with inconspicuous conidial scars, 0.5–1 µm wide. ***Chlamydospores*** rare, solitary or in chains, terminal, globose to pyriform, without or one-septate, pigmented, dark brown, smooth-, thick- walled. ***Sexual morph*** unknown.

##### Cardinal temperatures for growth on MEA after two weeks

**(mm).** Optimum at the range of 25 °C to 30 °C (10 to 14). No growth 4 °C and 35 °C.

##### Additional materials examined.

Thailand • Chiang Mai Province, Chai Prakan District, Nong Bua Subdistrict, endolichenic from the medulla of foliose lichen (*Parmotrema* sp.) on *Prunusdomestica*, 19°42'23"N, 99°1'32"E, elevation 1160 m, 2 June 2023, C. Senwanna and N. Suwannarach, living culture (LC05-3 = SDBR-CMU503).

##### Additional GenBank numbers.

*act* and *tef1* for SDBR-CMU502: PQ523738 and PQ523739; *tef1* for SDBR-CMU503: PQ523740.

##### Ecology and distribution.

Endolichenic fungi from the medulla of foliose lichen (*Parmotrema* sp.) in Thailand.

##### Notes.

Based on a blast search of the NCBI’s GenBank nucleotide database of the ITS sequence, *Atrokylindriopsisracemosospora* has the closest match with *At.setulosa* (strain HMAS245592; KP337330, ex-type) with 99.33% similarity and is similar to *Aciculomycesrestrictus* (strain FMR 18994; ON009870, ex-type) with 93.79% similarity (identities = 468/499, 10 gaps) and *Exophialasiamensis* (strain SDBR-CMU417; NR_184988, ex-type) with 92.39% similarity (identities = 583/631, 16 gaps). The closest matches using the LSU sequence are *At.setulosa* (strain HMAS245592; KP337329) with 100% similarity (identities = 548/548, 0 gap), *Ex.ramosa* (strain FMR 18632; ON009933, ex-type) with 98.97% similarity (identities = 865/874, 1 gap), and *Melanoctonatectonae* (strain MFLUCC 12-0389; NG_059687) with 98.43% similarity (identities = 879/893, 2 gaps). The closest matches using SSU sequence are *Ex.siamensis* (strain SDBR-CMU417; ON555826) with 99.53% similarity (identities = 1055/1060, 1 gap), *Caproniadactylotricha* (strain CBS 604.96; NG_062636) with 98.45% similarity (identities = 1082/1099, 1 gap), and *E.yunnanensis* (strain YMF1.06739, ex-type) with 98.31% similarity (identities = 1049/1067, 1 gap). The closest matches using the *tub2* sequence are *Biscogniauxiaarima* (strain YMJ 122; AY951672) with 93.08% similarity (identities = 121/130, 1 gap) and *B.mediterranea* (strain ISN9LDC31; OQ942633) with 92.37% similarity (identities = 121/131, 1 gap).

A combined multilocus-based phylogenetic analysis showed that both strains (SDBR-CMU502 and SDBR-CMU503) of *Atrokylindriopsisracemosospora* formed sister taxon to *At.setulosa* (HMAS245592, ex-type strain) with 100% ML and 1 PP statistical support, and also clustered with *Aciculomycesrestrictus* (FMR 18994, ex-type strain) with 95% ML and 1 PP statistical support (Fig. 1). The ITS and LSU nucleotide sequence comparisons reveal 3/580 and 0/548 base pair differences with *At.setulosa*, respectively. In contrast, a comparison of ITS, LSU, *tef1*, and *tub2* base pairs shows that *At.racemosospora* differs from *Ac.restrictus* by 34/494 bp of ITS, 8/770 bp of LSU, 38/158 bp of *tef1*, and 108/430 bp of *tub2*. The morphology of *Atrokylindriopsis* differs from that of *Aciculomyces* in having unbranched, macronematous conidiophores, monophialidic conidiogenous cells, pigmented, septate, setulate conidia (Ma et al. 2015; Torres-Garcia et al. 2023). *Atrokylindriopsisracemosospora* shares similar features, including sympodial proliferation, denticulate conidiogenous cells, and aseptate, obovoid or subglobose conidia, with species *Ac.restrictus* (Torres-Garcia et al. 2023), but *Ac.restrictus* has longer conidiophores (10–153 × 1.4–2.9 µm *vs* 19–105.5 × 1.5–2.5 µm) and smaller conidia (2.5–5 × 1.8–3.3 µm *vs* 2–4 × 1.5–2.5 µm). Moreover, the conidiogenous cells and conidia formation of *At.racemosospora* is more abundant than that of *Ac.restrictus*, and the conidial scars are not conspicuous. In addition, *At.setulosa* differs from *At.racemosospora* by its larger, cylindrical to clavate or rounded-cuboid conidia, which have 4–5 longitudinal eusepta and are attached to the conidiogenous locus at the midpoint of their long side, appearing to form a ‘T’ (Ma et al. 2015). These morphological characteristics clearly distinguish *At.racemosospora* from *Ac.restrictus* and *At.setulosa*.

#### ﻿ Phialophora chinensis

Taxon classificationFungiChaetothyrialesHerpotrichiellaceae

Ya L. Li, de Hoog & R.Y. Li, in Li, Xiao, de Hoog Wang, Wan, Yu, Liu & Li, Persoonia 38: 11 (2016)

32967B0D-AB3F-5325-ABBD-917294DD0695

815345

Fig. 3

##### Cultural characteristics.

Colonies on different agar media were incubated in the dark at 25 °C for 1 months; colonies flat, irregular, with edge entire, velvety; on PDA (39 to 44 mm in diameter) surface grayish brown, dark brown to black at the margin, reverse olivaceous black; on MEA surface Blackish Grey, black at the margin, reverse olivaceous black; on MEA (49 to 53 mm in diameter) surface Blackish Grey, black at the margin, reverse olivaceous black; on OA (69 to 74 mm in diameter) surface grayish brown, reverse olivaceous black; on PCA (46 to 55 mm in diameter) surface and reverse grayish brown, dark brown at the margin, reverse olivaceous black, sporulation absent; on CMD (42 to 50 mm in diameter) surface and reverse brownish gray; and on CMA (33 to 41 mm in diameter) surface and reverse brownish grey, sporulation absent. ***Asexual morph in vitro*** dematiaceous hyphomycetes. ***Hyphae*** 1–3.4 µm wide, subhyaline to light brown, simple to branched, septate, smooth-, thin-walled, coiling, anastomosis observed. ***Conidiophores*** (4–)5.5–31(–45.3) × (2–)3–4.3(–4.8) µm (*x̄* = 17 × 3.6, *n* = 55), micro- or semi-macronematous, straight, simples or poorly branched, septate, slightly constricted at septa, smooth; micronematous conidiophores consisting in conidiogenous cells growing directly from vegetative hyphae, lateral or terminal. ***Phialides*** 4–11(–15.5) × 2.3–4(–5) µm (*x̄* = 8.3 × 3.3, *n* = 75), regularly flask-shaped to elongate-ampulliform or subulate, with an apical conspicuous collarette; collarettes (1.6–)2–3.9(–4.8) × (1.6–)2–3.9(–4.2) µm (*x̄* = 3 × 3, *n* = 65), usually funnel-shaped, darker than the rest of the phialide. ***Conidia*** 2–4(–5) × 1.6–2.7(–3.9) µm (*x̄* = 3.3 × 2.2, *n* = 75), hyaline to subhyaline, mostly broadly ellipsoidal, more rarely obovoidal, smooth-walled. ***Chlamydospores*** 9.5–20(–23) µm wide, abundantly produced in aerial hyphae, mostly intercalary, solitary or in branched chains, sub globose to ellipsoidal or barrel-shaped, pigmented, light to dark brown, smooth-, thick-, dark-walled, without or one-septate, constricted near the septa. ***Sexual morph*** unknown.

**Figure 3.**
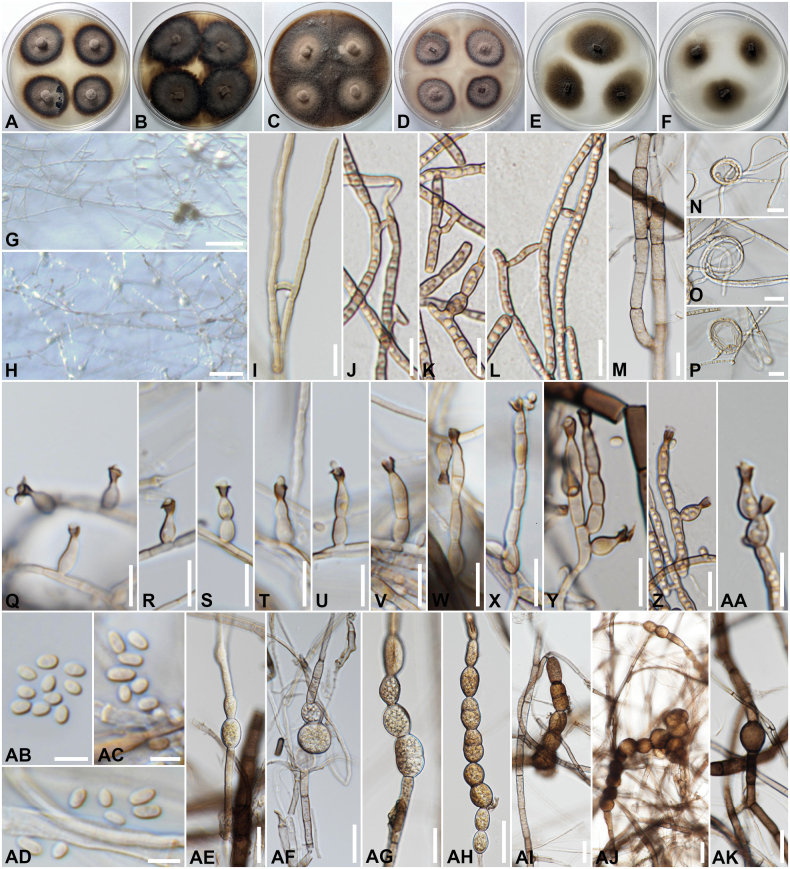
*Phialophorachinensis* (SDBR-CMU506, new host record). A–F. Colonies on PDA, MEA, OA, PCA, CMD and CMA, respectively, after 30 d at 25 °C; G. Conidiophores sporulating on CMA; H. Conidiophores sporulating on PCA; I–M. Hyphal anastomosis; N–P. Hyphal coil; Q–AA. Phialides at different stages of development; AB–AD. Conidia; AE–AK. Chlamydospores (intercalary and chains formation). Scale bars: 100 µm (G, H); 10 µm (I–AA); 5 µm (AB–AD); 20 µm (AE–AK).

##### Cardinal temperatures for growth on MEA after two weeks

**(mm).** Optimum at the range of 25 °C to 30 °C (19 to 24), maximum 35 °C (17 to 19). No growth 4 °C.

##### Materials examined.

Thailand • Chiang Mai Province: Mueang Chiang Mai District, Suthep Subdistrict, endolichenic from the medulla of foliose lichen (*Parmotrema* sp.) on unidentified tree trunk, 18°48'27"N, 98°56'37.7"E, elevation 343 m, 26 June 2023, C. Senwanna, living culture: LC10-10 = SDBR-CMU504, LC10-11 = SDBR-CMU505, and LC10-17 = SDBR-CMU506.

##### Ecology and distribution.

Endolichenic fungi from the medulla of foliose lichen (*Parmotrema* sp.) in Thailand (this study); pathogenic as chromoblastomycosis in human in China and Mexico (Li et al. 2017; Ahmed et al. 2021), as fungal keratitis in India and USA (Ply et al. 2023; Mitra et al. 2024), as phaeohyphomycosis in France (Pruvot et al. 2023); saprobic from bamboo in China, from the environment in Japan, from plant materials in Brazil and China, and soil in China and from wheat straw in Brazil (Li et al. 2017).

##### Notes.

A BLASTn search using ITS and *tub2* sequence data in NCBI has revealed sequence similarities of 98.98–99.83% and 98.46–99.78% between our strains (SDBR-CMU504, SDBR-CMU505, and SDBR-CMU506) and *Phialophorachinensis* strains. While the closest matches using the LSU sequence are *P.ellipsoidea* (strain MUCL 9768; AF050282) with 99.89% similarity (identities = 885/886, no gap), *P.macrospora* (strain MUCL 15541; EU514701), and *P.americana* (strain UAMH 10872; EU514691) with 99.77% similarity (identities = 864/866, no gap). The closest matches using the SSU sequence are *Caproniasemiimmersa* (strain UAMH 10872; JN941209) with 99.71% similarity (identities = 1018/1021, 1 gap), *P.verrucosa* (strain AFTOL-ID 670; EF413614) with 99.60% similarity (identities = 992/996, 2 gaps), and *P.americana* (strain CBS 840.69; AY554291) with 99.33% similarity (identities = 1033/1040, 4 gaps).

In our multigene phylogenetic study, strains SDBR-CMU504, SDBR-CMU505, and SDBR-CMU506 form a clade with close affinity to *P.chinensis* (Fig. 1). However, the species segregation within the taxa is not discrete in a multigene phylogeny. Comparing the ITS, LSU, SSU, and *tub2* regions between our strains and CBS 140326 (type strain), only 1 bp difference was found in the ITS, 3 bp difference in LSU and SSU, and no base pair differences in the *tub2*. The morphology of our strains resembles the species description of *P.chinensis* provided by Li et al. (2017), except for the appearance of the conidiophores and chlamydospores and the lack of budding conidia. Although our strains have shorter conidia compared to *P.chinensis* CBS 140326, the holotype isolated from skin lesions of a human chromoblastomycosis patient (2–5 × 1.6–3.9 μm *vs* 3–6 × 2.0–5.5 μm), the detail of other structures (i.e., collarette, conidiophore, conidiogenous cells and chlamydospores) was not mentioned. Hence, an updated morphology for the species *P.chinensis* is provided.

#### ﻿ Veronaea endolichena

Taxon classificationFungiChaetothyrialesHerpotrichiellaceae

Senwanna, J. Kumla & N. Suwannar. sp. nov.

8CD13133-4FCA-53A2-9B7C-4643DE7A4941

856324

Fig. 4

##### Etymology.

Refers to the host substrate.

##### Type.

Thailand • Chiang Mai Province: Mueang Chiang Mai District, Suthep Subdistrict, endolichenic from the medulla of foliose lichen (*Parmotrema* sp.) on unidentified tree trunk, 18°48'27"N, 98°56'37.7"E, elevation 343 m, 26 June 2023, C. Senwanna, CMUB40068 (***Holotype***, preserved in a metabolically inactive state. ***Ex-type*** lliving culture LC10-9-2 = SDBR-CMU507).

**Figure 4.**
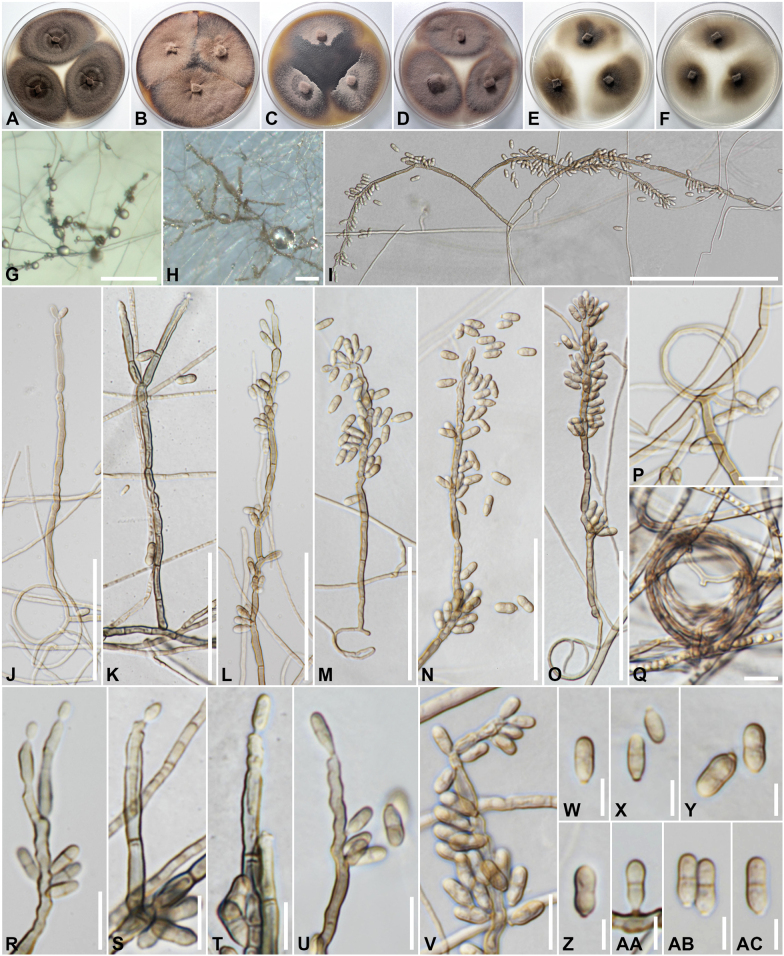
*Veronaeaendolichena* (SDBR-CMU507, ex-type). A–F. Colonies on PDA, MEA, OA, PCA, CMD and CMA, respectively, after 14 d at 25 °C G. Conidiophores sporulating on PCA; H. Conidiophores sporulating on CMA; I–O. Conidiophores at different stages of development; P–Q. Hyphal coil; R–Y. Conidiophores with conidiogenous loci; W–A.C Conidia. Scale bars: 100 µm (G–I); 50 µm (J–O); 10 µm (P–V); 5 µm (W–AC).

##### Cultural characteristics.

Colonies on different agar media were incubated in the dark at 25 °C for 2 weeks; colonies flat, irregular, with edge entire, velvety; on PDA (63 to 67 mm in diameter) surface grayish brown, dark at the middle and margin, reverse olivaceous black; on MEA (42 to 59 mm in diameter) surface brownish gray, reverse olivaceous black, producing dark brown pigment in agar; on OA (57 to 60 mm in diameter) surface brownish gray, reverse olivaceous black; on PCA (61 to 63 mm in diameter) surface and reverse brownish gray, reverse olivaceous black, sporulation absent; on CMD (54 to 55 mm in diameter) surface and reverse grayish brown; and on CMA (51 to 58 mm in diameter) surface and reverse brownish gray, sporulation absent. ***Asexual morph in vitro*** dematiaceous hyphomycetes. ***Hyphae*** 1–3.8 µm wide, subhyaline to light brown, simple to branched, septate, smooth, thin-walled, coiling observed. ***Conidiophores*** (23–)38–200(–264) × 2.3–3.7(–4.9) µm (*x̄* = 118.6 × 3.1, *n* = 25), macronematous, mononematous, erect, straight or slightly flexuous, branched, septate, cylindrical, rough-, thick-walled, light to dark brown. ***Conidiogenous cells*** (7.5–)12–102(–136.5) × 2.1–3.5(–3.9) µm (*x̄* = 53.5 × 2.9, *n* = 30), integrated, polyblastic, terminal to mostly intercalary, cylindrical, pale brown to brown, fertile parts subhyaline, rachis with crowded, flat to slightly prominent, unthickened scars. ***Conidia*** (5–)6.5–11(–13.5) × 2.5–4(–4.6) µm (*x̄* = 8.5 × 3.3, *n* = 90), solitary, cylindrical to pyriform, rounded at apex, truncate at base, lower cell longer and wider than upper one, with a prominent scar, 0.8–1.9 µm wide, pale brown, without or one median septate, constricted at the septa, smooth-walled. ***Chlamydospores*** absent. ***Sexual morph*** unknown.

##### Cardinal temperatures for growth on MEA after two weeks

**(mm).** Optimum 25 °C (35 to 40), maximum 30 °C (20 to 21). No growth 4 °C and 35 °C.

##### Additional materials examined.

Thailand • Chiang Mai Province, Mueang Chiang Mai District, Suthep Subdistrict, endolichenic from the medulla of foliose lichen (*Parmotrema* sp.) on unidentified tree trunk, 26 June 2023, 18°48'27"N, 98°56'37.7"E, elevation 343 m, C. Senwanna, living culture LC10-2 = SDBR-CMU508 and LC10-12 = SDBR-CMU509.

##### Ecology and distribution.

Endolichenic fungi from the medulla of foliose lichen (*Parmotrema* sp.) in Thailand.

##### Notes.

The closet match in a BLASTn search in GenBank with the ITS, LSU, and *tub2* sequence of *V.endolichena* had highest similarity to *V.botryosa* strain GZCC:19-0557 (OP377853, with 99.65%, identities = 577/579, 2 gaps), strain CBS 127264 (MH875936, with 100%, identities = 908/908, no gap) and strain CBS 121506 (JN112502, with 93.61%, identities = 381/407, no gap), respectively. While the match using SSU sequence are *Ex.yunnanensis* (strain YMFT 1.06739; MZ781222, holotype) with 99.72% (identities = 1053/1056, no gap), *Thysanoreaamniculi* (strain SGT69-1; OP378033, holotype) with 99.71% (identities = 1019/1022, no gap) and *Ex.aquamarine* (strain IMP-BG-H0001; MH813287) with 99.63% (identities = 1072/1076, no gap). Multigene phylogenetic analysis of the combined dataset revealed that three strains (SDBR-CMU507, SDBR-CMU508, and SDBR-CMU509) of *V.endolichena* clustered as a sister taxon to *V.botryosa*; however, the ITS and *tub2* sequences of *V.endolichena* differ from *V.botryose* in 6 bp/610 and 25/393 bp, respectively. Morphologically, *V.endolichena* differs from *V.botryosa* in longer conidiophores, the number of septa (0–1 *vs* 0–3 septa) and size of the conidial scar (0.8–1.6 *vs* 0.5 µm) (Table 2) (Arzanlou et al. 2007, Yang et al. 2023).

**Table 2.** *Veronaea* species with a synopsis of the characteristics and relevant references.

*Veronaea* species	Conidiophore	Conidiogenous cells	Conidia	Sequence data	Reference
*V.aquatica*	Up to 280 × 2.5–4 μm. Erect, the lower part is usually straight, and the upper half is usually flexuous, usually loosely branched, sometimes geniculate, pale brown to dark brown, smooth-walled.	(3–)10–30 × 2–3.5 µm. Terminally integrated, polyblastic, occasionally intercalary, cylindrical, variable in length, pale brown, later often becoming septate, fertile part subhyaline, wide at the basal part, rachis with crowded, flat to slightly prominent, faintly pigmented; scars flat, slightly pigmented, not thickened, about 0.65 μm diam.	6–11(–12) × 2.5–3.5(–4.0) µm. Solitary, smooth, cylindrical to subpyriform and some subclavate, pale brown, most medially 1-spetate, rarely 0 or 2-septate, often constricted at the septum and the colour septum middle brown and the conidia with a round apex and truncate base; with a faintly darkened, unthickened hilum, about 0.5–0.9 μm diam.	Present	Chandrasiri et al. (2021)
*V.botryosa*	Up to 250 × 2–3 μm. Erect, straight or flexuose, unbranched or occasionally loosely branched, sometimes geniculate, pale brown to olivaceous brown, smooth-walled.	10–100 µm long. Terminal, occasionally intercalary, cylindrical, pale brown, later often becoming septate, fertile part subhyaline, often as wide as the basal part, rachis with crowded, flat to slightly prominent, faintly pigmented, unthickened scars.	(3–)6.5–8.5(–12) × (1.5–)2–2.5(–3) µm Solitary, smooth, cylindrical to pyriform, rounded at the apex and truncate at the base, pale brown, 1(–2)-septate, with a faintly darkened, unthickened hilum, about 0.5 µm diam.	Present	Arzanlou et al. (2007)
*V.caricis*	Up to 550 × 4–6 μm. Erect, straight or flexuose, sometimes swollen at the base to 10-14 µm, with numerous scars towards the apex, unbranched or occasionally loosely branched, sometimes geniculate, mid to dark brown, smooth-walled.	No information available	20–25 × 6–7 μm. Straight, fusiform or obclavate, 1 septate, hyaline or subhyaline, verruculose	Absent	Ellis (1976)
*V.carlinae*	Up to 130 × 2–4 μm. Simple or loosely branched, straight or flexuous, septate, pale brown, smooth, with numerous scars.	No information available	20–25 × 6–7 μm. Cylindrical, rounded at the apex and conico-truncate at the base, or fusiform, 1–3 septate, pale brown, smooth	Absent	Ellis (1976)
*V.compacta*	Up to 60 µm long. Slightly differentiated from vegetative hyphae, lateral or occasionally terminal, often wider than the supporting hypha, up to 4 µm wide, unbranched or branched at acute angles, with 1–3 additional septa, cells often inflated and flask-shaped, pale brown.	Up to 10 µm long. Terminal, occasionally intercalary, variable in length, cylindrical to doliiform or flask-shaped, with hardly prominent denticles, pale brown; scars flat, slightly pigmented, not thickened, about 0.5 µm diam.	(4–)6–7(–9) × 2–3 µm. Solitary, ellipsoidal to ovoid, 0–1(–2)-septate, often constricted at the septa, with a round apex and truncate base, pale brown, smooth, thin-walled; hilum prominent, slightly darkened, unthickened, about 0.5 µm diam.	Present	Arzanlou et al. (2007)
*V.coprophila*	Up to 350 × 3–4.5 µm. Straight or flexuous, septate, mid to dark brown, paler towards the apex where there are a number of small, flat scars.	No information available	6–12 × 3–5 µm. Straight, cylindrical, rounded at the apex, conico-truncate at the base or ellipsoidal, 1–2 septate, smooth, pale brown.	Absent	Ellis (1976)
*V.endolichena*	(23–)38–200(–264) × 2.3–3.7(–4.9) µm. Erect, straight or slightly flexuous, branched, septate, cylindrical, light to dark brown, rough-, thick-walled.	(7.5–)12–102(–136.5) × 2.1–3.5(–3.9) µm. Integrated, polyblastic, terminal to mostly intercalary, cylindrical, pale brown to brown, fertile parts subhyaline, rachis with crowded, flat to slightly prominent, unthickened scars.	(5–)6.5–11(–13.5) × 2.5–4(–4.6) µm. Solitary, cylindrical to pyriform, rounded at apex, truncate at base, lower cell longer and wider than upper one, with a prominent scar, 0.8–1.9 µm wide, pale brown, without or one median septate, constricted at the septa, smooth-walled.	Present	This study
*V.ficina*	30–120 × 2.5–4.75 µm. Superficial, arising singly as lateral or terminal branches from external hyphae ranging from macronematous to mononematous, erect, straight to flexuous or slightly curved, smooth-walled, unbranched, septate with brown base and paler apex.	Integrated, polyblastic, terminal to predominantly intercalary, sympodial, cylindrical, cicatrized with thickened scars.	4.5–18.5 × 5.5 μm. Holoblastic, dry, simple, not catenate, finely verruculose, obovoidal, base obconicotruncate, apex rounded, 0–3-septate, pale brown.	Absent	Kharwar and Singh (2004)
*V.filicina*	400–800 × 2–2.5 µm. Base rarely swollen, rarely branched, sparsely septate, at first thin-walled, roughened walls, olivaceous then to brown.	No information available	6–13.5 × 3–4 μm. Elliptical or pyriform, not constricted at septa, 1–3- septate, olivaceous or fuscous.	Absent	Dingley (1972)
*V.gobica*	Up to 350 × 1.5–2.5 µm. Straight or flexuous, septate, smooth, with numerous minute scars towards the apex, brown.	No information available	4–6.5 × 2–3.5 µm. Ellipsoid, smooth, Subhyaline or pale brown	Absent	Pan and Zhang (2009)
*V.grewiicola*	42–104 × 2.5–6.3 µm. Sometimes curved, smoothwalled, branched, 2–5 septa, pale brown to brown base and paler apex.	Integrated, polyblastic, terminal to mostly intercalary, sympodial and cicatrized with thickened scars.	7.3–19.5 × 2.6–7.5 µm. Holoblastic, dry, simple, non-catenate, finely verruculose, sometimes constricted at the septa, hilum thickened with truncate base and rounded apex, 0–2(–3) septate subhyaline or pale brown	Absent	Kharwar and Singh (2004)
*V.hedychii*	19.5–86.5 × 2.5–3.5 µm. Sometimes with an inflated base up to 7 µm diam., straight to slightly curved, unbranched, 1–6 septate, golden brown to brown, paler towards the apices, smooth.	10–40 × 1.5–2.5 µm. Terminal, holoblastic, sometimes intercalary, densely and minute cicatrized, scars thickened, 1 µm wide.	5–10 × 2–4 µm. Ellipsoid, cylindrical or obovoid, base obconic truncate, apex rounded, 0–1 septate, sometimes slightly constricted at the septum, subhyaline to pale brown, smooth, hila thickened, somewhat darkened, 1 µm diam.	Absent	Soares and Barreto (2008)
*V.hippocrateae*	27–123 × 2–4 µm. Straight to slightly curved, unbranched, paler towards the apices, 1–6 septate, smooth, brown to dark brown.	No information available	4–18.5 × 1.5–3.5 µm, Solitary, non-catenate, smooth-walled, obconico base, apex rounded, 0–1-septate, cylindric to obclavate, slightly curved, subhyaline to pale brown	Absent	Kharwar and Singh (2004)
*V.japonica*	Up to 65 × 2–3 µm. Slightly differentiated from aerial vegetative hyphae, lateral, or terminal, often wider than the supporting hypha, unbranched or occasionally branched, pale brown, thin-walled, smooth, with 1–3 additional septa.	Up to 15 µm long. Terminal, occasionally intercalary, variable in length, pale brown, cylindrical to clavate, with hardly prominent denticles; scars flat, slightly pigmented, not thickened, about 0.5 µm diam.	(6–)7–8(–10) × 2–2.5(–4) µm. Solitary, pale brown, smooth, thin-walled, ellipsoidal to ovoid, (0–)1-septate, often constricted at the septum, with a round apex and truncate base; hilum unthickened but slightly darkened, about 1 µm diam.	Present	Arzanlou et al. (2007)
*V.latispora*	Up to 90 × 1.5–2.5 µm. Straight or slightly curved, septate, smooth, with numerous minute scars at the upper parts, brown.	No information available	7.5–9.5 × 3–5 Broadly obovoid, smooth, aseptate, subhyaline to pale brown.	Absent	Pan and Zhang (2009)
*V.oblongispora*	Up to 320 × 3–5 µm. Solitary or sometimes in fascicles, smooth, bulbous towards the base, pale brown to brown.	No information available	7–8 × 4–5 µm. Oblong, smooth, aseptate, rather thick-walled, obtuse at the apex, subhyaline to pale brown.	Absent	Morgan-Jones (1982)
*V.polyconidia*	1125–1515 × 45–70 μm. Straight or slightly flexuous, unbranched, solitary, cylindrical, rough-walled, thick-walled, brown to dark brown.	Polyblastic, terminal, cylindrical, rachis with crowded, flat to slightly prominent, pale brown, fertile parts subhyaline.	11–16 × 3–5 μm. Solitary and smooth, cylindrical to pyriform, rounded at apex and truncate at base, pale brown, 1–3-septate (mostly 3), often constricted and medium brown at septa.	Present	Su et al. (2023)
*V.queenslandica*	No information available	No information available	No information available	Absent	Matsushima (1989); Index Fungorum (2025)
*V.smilacis*	70–165 (–90–125) × 3.5–5.5 μm. Solitary, thick-walled, smooth, up to 12-septa, brown.	No information available	21.5–32.5 × 2–5 μm. Solitary, cylindrical to slightly obclavate, 1–12 septate, smooth-walled, subhyaline to pale olivaceous.	Absent	Singh et al. (1981)
*V.tectonae*	Up to 200 × 3.6 μm. Solitary smooth, up to 10-septa, pale brown.	No information available	11 × 3.75 μm. Solitary, simple, smooth, usually 1-septa, prominent scars at the base.	Absent	Kamal (1980)
*V.thylacospermi*	35–100 × 3–4 μm. Simple, smooth, brown, 1–3(–8) septate, basal cell inflated 6–10 µm wide, fertile part taller than basal part, forming slightly flexuous rachis with scattered, hyaline and small, apically pointed denticle-like.	Conidiogenous loci 0.3 µm high, 0.5 µm wide.	12–14 × 3–4 μm. Two-celled, lower cell longer and wider than upper one, wall slightly verruculose, hyaline to pale brown.	Absent	Chlebicki (2009)

## ﻿Discussion

Several studies have revealed that lichen thalli host diverse microbial communities, including fungi (Oh et al. 2020; Yang et al. 2021, 2022; Diederich et al. 2022, 2024; Miral et al. 2022; Cometto et al. 2023; Si et al. 2023). Endolichenic fungi that reside inside lichens without causing any symptoms not only thrive in harsh environments but also generate unique pharmacological activities and bioactive compounds similar to those of lichens (Kellogg and Raja 2017; Singh et al. 2017; Suryanarayanan and Thirunavukkarasu 2017; Moreno et al. 2018; Agrawal et al. 2020; Yang et al. 2021; Zhang et al. 2021, 2024; Perera et al. 2022; Cometto et al. 2023). The presence of endolichenic fungi within lichen thalli suggests a symbiotic or commensal relationship, whereby these fungi enhance lichen health and resilience by producing protective secondary metabolites and potentially participating in organic matter decomposition and nutrient recycling within the thallus or its substrate (Tsurykau and Etayo 2017; Muggia and Grube 2018; Diederich et al. 2024). These functional roles may help explain the ecological success of lichens in diverse and often extreme environments, highlighting the significance of endolichenic fungi within lichen ecosystems and underscoring the need for further studies to elucidate their functions. Most endolichenic fungi have been documented from tropical areas, primarily China and India, and it is anticipated that research in other areas will yield a wealth of new species, biological compounds, and ecological data (Kellogg and Raja 2017; Suryanarayanan et al. 2017; Agrawal et al. 2020; Si et al. 2023). Recent publications have identified eight new Chaetothyriales species (*Cladophialophoraendolichena*, *C.guttulate*, *C.haematommatis*, *C.heterodermiae*, *C.holosericea*, *C.olivacea*, *C.yunnanensis*, and *Paracladophialophoralichenicola*) and a new genus, *Intumescentia* in Teratosphaeriaceae, based on phylogeny and morphology and other known species in Eurotiomycetes, Dothideomycetes, and Sordariomycetes from lichen thalli (Chang et al. 2023; Cometto et al. 2023, 2024; Si et al. 2023; Diederich et al. 2024). Likewise, studies on secondary metabolites and their activities, isolated from endolichenic fungi growing in various environments, are increasing (Zhang et al. 2021; Toure et al. 2022; Varlı et al. 2022, 2023; Zhang et al. 2024). Therefore, the investigation of new ecosystems, particularly in tropical countries, is likely to reveal novel endolichenic fungi with unique potential, thereby enhancing scientific knowledge. This research employed a culture-based approach, focusing on endolichenic fungi, and is part of an ongoing study examining the diversity of endolichenic fungi in northern Thailand. Three endolichenic fungal species in Herpotrichiellaceae were identified based on morphology and phylogenetic evidence from combined datasets of ITS, LSU, SSU, and *tub2* genes.

*Atrokylindriopsis* was established by Ma et al. (2015) to accommodate the single species *At.setulosa*. *Atrokylindriopsissetulosa* was reported as saprobic on dead branches of an unidentified broadleaf tree and was placed in Chaetothyriales without a family assigned, based on a BLASTn search using ITS and LSU sequence data from NCBI and morphological comparisons (Ma et al. 2015). Wijayawardene et al. (2020) considered this genus to be in the Chaetothyriales, but later Quan et al. (2020) assigned it to the Herpotrichiellaceae. Morphologically, the pigmented, septate, setulate conidia are a unique feature of *Atrokylindriopsis*, distinguishing it from other members of Herpotrichiellaceae. In the present phylogenetic tree (Fig. 1), the new species, *At.racemosospora*, is closely related to *At.setulosa*. *Atrokylindriopsisracemosospora* exhibits morphological differences from *At.setulosa*, as mentioned above. Based on morphology, *At.racemosospora* might represent a different genus from *Atrokylindriopsis*. The holotype (HMAS245592) and dried culture specimens (HSAUP H4560) of *At.setulosa* were not reexamined, as they had been lost. Consequently, it was not possible to reassess the morphology. Due to the similarity in the ITS and LSU sequences between *At.racemosospora* and *At.setulosa*, along with the unavailability of protein-coding gene sequence data of *At.setulosa*, it was deemed insufficient evidence to justify separating them into different genera. Since the molecular data were unclear in resolving the relationships between *At.setulosa* and our specimens, we tentatively classified our strains as a new species within *Atrokylindriopsis*. Additionally, the examination of newly collected specimens, morphology, and molecular data regarding protein-coding genes of *At.setulosa* requires further study. It is worth noting that *At.racemosospora* is similar to *Aciculomyces* and *Petriomyces* in having sympodial conidiogenous cells that produce conidia from short denticles and obovoid, aseptate conidia (Thitla et al. 2023; Torres-Garcia et al. 2023). Based on morphological characters and multi-gene phylogenetic analysis, *Aciculomyces* was established by Torres-Garcia et al. (2023) to accommodate the single species *Ac.restrictus*, collected from fluvial sediments, while *Petriomyces*, typified by *P.obovoidisporus*, was isolated from sandstone in a natural forest (Thitla et al. 2023). Moreover, *At.racemosospora* can be distinguished from these genera by the abundance of sporulation, inconspicuous conidial scars, and the phylogenetic analysis, which also segregates these genera.

*Phialophora* is known as a group of black yeasts and their relatives, which have been reported as pathogens in humans and animals, as well as saprobic and endophytic in plant materials and soil (Schol-Schwarz 1970; Untereiner et al. 2008; Liu et al. 2013; Li et al. 2017). The genus is typified by *P.verrucosa* and characterized by pigmented hyphae, solitary or aggregated conidiophores, cylindrical to flask-shaped phialides bearing conspicuous, darkly pigmented collarettes, and ovoid to cylindrical, aseptate conidia (Medlar 1915; Schol-Schwarz 1970; Gams 2000; Untereiner et al. 2008). Currently, 38 epithets are listed for *Phialophora* in Index Fungorum (2025). Morphology alone is insufficient for species identification within *Phialophora*; moreover, molecular data have demonstrated significant genetic variation within the species complex, now referred to as the *P.verrucosa* species complex (Li et al. 2017). Several species have been transferred to other genera, such as *Chloridium*, *Cyphellophora*, *Entimomentora*, *Hyphodiscus*, *Lasiosphaeris*, and *Rhopalophora*, based on morpho-molecular analyses (Bogale et al. 2010; Réblová et al. 2011, 2013, 2017; Johnston et al. 2014; Untereiner et al. 2019). Although many sequences of *Phialophora* are available in GenBank, in our pre-analyses, *P.asteris*, P.asterisf.sp.helianthi, *P.atrovirens*, *P.avicenniae*, *P.cinerescens*, *P.cyclaminis*, *P.dancoi*, *P.foetens*, *P.intermedia*, *P.japonica*, and *P.mustea* were grouped outside of the clade comprising members of Herpotrichiellaceae. Therefore, we excluded these sequences from our analysis. Our molecular phylogenetic tree indicated that our three strains formed a separate clade within the *P.chinensis* clade, with representative strains of *P.chinensis* separated into two subclades, supported by 86% ML and 1 PP statistical support (Fig. 1). Sequence comparisons of ITS, LSU, SSU, and *tub2* genes between our strains and representative strains from two sub-clades indicate that our strains are congeneric. The conidial size of our strains aligns well with *P.chinensis*; however, the dematiaceous hyphomycetes of our strains cannot be directly compared with the type, as Li et al. (2017) did not provide a detailed description of conidiophores, conidiogenous cells, collarettes, and chlamydospores. According to Li et al. (2017), *P.chinensis* has been isolated from human patients, leaves, soil, wheat straw, and wood, which may account for morphological variation within the species. Based on the available data, we identified our strains as *P.chinensis*, which were collected as endolichenic fungi associated with foliose lichens in Thailand. *Phialophora* identification was initially based on morphology and molecular phylogeny (Yan et al. 1995; de Hoog et al. 1999; Untereiner et al. 2008). Although similar conidia can be observed among *Phialophora* species, genetic variation in the ITS, LSU, and *tub2* gene regions can be used to distinguish species. However, since most available sequences of *P.chinensis* contain only three gene regions and excluded species contain only the ITS gene region, species delineation based on phylogeny remains uncertain. Therefore, more informative genes (i.e., *act*, *tef1*, and *rpb2*) and additional collections may help clarify species placement and reveal the possibility of a species complex.

*Veronaea* was introduced by Ciferri and Montemartini (1957) and is typified by *V.botryose*. There are 20 epithets recorded in Index Fungorum (2025), but only five species have been identified with DNA sequence data. Based on multi-gene analyses and morphological comparisons, *V.constricta* (CBS 572.90, type strain) has been considered a synonym of *V.botryosa* (Su et al. 2023; Yang et al. 2023). In our phylogenetic tree, the *Veronaea* species clusters as a monophyletic clade (Fig. 1), maintaining a stable position within Herpotrichiellaceae, which concurs with the findings of Su et al. (2023) and Yang et al. (2023). *Veronaeaendolichena* is introduced based on phylogenetic analysis and morphological data. A morphological comparison between *V.endolichena* and other *Veronaea* species is shown in Table 2. *Veronaeaendolichena* is similar to *V.caricis*, *V.compacta*, *V.hedychii*, *V.hippocrateae*, *V.japonica*, *V.tectonae*, and *V.thylacospermi* in having 0–1-septate conidia. However, the conidia of *V.compacta* (4–9 × 2.5–4 µm), *V.hedychii* (5–10 × 2–4 µm), *V.japonica* (6–10 × 2–4 µm) and *V.tectonae* (11 × 3.75 µm) are shorter than those of *V.endolichena* (5–13.5 × 2.5–4.6 µm) (Ellis 1976; Kamal 1980; Kharwar and Singh 2004; Arzanlou et al. 2007; Soares and Barreto 2008; Chlebicki 2009). *Veronaeacaricis* (20–25 × 6–7 µm) and *V.hippocrateae* (4–18.5 × 1.5–3.5 µm) have longer conidia than *V.endolichena* (Ellis 1976; Kharwar and Singh 2004). *Veronaeaendolichena* (5–13.5 × 2.5–4.6 µm) is closely related to *V.thylacospermi* (12–14 × 3–4 µm); however, *V.endolichena* has longer conidiophores (23–264 × 2.3–4.9 µm *vs* 35–100 × 3–4 µm) and cylindrical to pyriform, smooth-walled conidia (Chlebicki 2009). Based on both morphological characteristics and multigene analyses, a novel species is introduced. Phylogenetically, *E.brunnea* (CBS 587.66) also clustered within the *Veronaea* clade (Fig. 1), which concurred with the results conducted by Costa et al. (2023) and Torres-Garcia et al. (2023). *Exophialabrunnea* differs from *Veronaea* in its conidiophores and phialides (Papendorf 1969; de Hoog et al. 2011); therefore, it was maintained as a separate species pending further investigation. Most species of *Veronaea* are known to be saprobes on various hosts worldwide (Arzanlou et al. 2007; Soto et al. 2017; Chandrasiri et al. 2021; Su et al. 2023; Yang et al. 2023). Since *Veronaea* exhibits overlapping characteristics, species delimitation based solely on morphology is challenging (Su et al. 2023); therefore, additional sampling is necessary for further investigation.

Our phylogenetic analyses indicated that *Capronia*, *Cladophialophora*, *Exophiala*, *Fonsecaea*, and *Rhinocladiella* are polyphyletic within the family Herpotrichiellaceae, which is concurred with Teixeira et al. (2017), Thitla et al. (2023), and Torres-Garcia et al. (2023). Whereas *Aciculomyces*, *Aculeata*, *Atrokylindriopsis*, *Marinophialophora*, *Melanoctona*, *Petriomyces*, *Phaeoannellomyces*, *Phialophora*, *Thysanorea*, *Uncispora*, *Valentiella*, and *Veronaea* represent a monophyletic group. Considering the asexual morph resemblance together with molecular support, Hernández-Restrepo et al. (2020) treat most *Minimelanolocus* species under *Thysanorea*. Although several species of *Minimelanolocus* have been recently reported based on morphological characteristics, these species lack sequence data, including the type species (Costa et al. 2018; Barreto et al. 2024). *Pleomelogramma* is also considered a doubtful genus in Herpotrichiellaceae, pending further studies due to the lack of sequence data for the type species. (Quan et al. 2020). Based on molecular analyses with distinctive ecological trends, de Hoog et al. (2011) defined the members of this family into six clades: *bantiana*-, *carrionii*-, *salmonis*-, *europaea*-, *dermatitidis*-, and *jeanselmei*-clades. With morphology and multi-gene phylogeny analyses, the species in the europaea clade were assigned as members of Cyphellophoraceae (Réblová et al. 2013). According to Wijayawardene et al. (2022), *Brycekendrickomyces*, *Metulocladosporiella*, *Neosorocybe*, and *Sorocybe* had previously been classified within Herpotrichiellaceae. However, the phylogenetic position of *Brycekendrickomyces* and *Metulocladosporiella* was presented in Trichomeriaceae (Quan et al. 2020, 2024). The ITS and LSU phylogenetic analyses by Quan et al. (2024) revealed that *Neosorocybe* and *Sorocybe* are separate from the Herpotrichiellaceae clade, forming a sister clade to the Epibryaceae clade in Chaetothyriales. Later, Thitla et al. 2023 treated both genera in Chaetothyriales*incertae sedis*.

Munk (1953) defined the characteristics of anamorphic Herpotrichiellaceae as dematiaceous hyphomycetes (black yeast), typified by proliferating, percurrently conidiogenous cells, conidiogenesis with holoblastic conidia produced from minute pegs or denticles, and unicellular conidia often held in chains. It is worth noting that several obligate lichenicolous Herpotrichiellaceae genera, such as *Cladophialophora*, have been found on lichen thalli or apothecia. These species differ from others in forming minute conidiophores in sporodochia, and in some species, two distinct types of conidia are observed (Diederich et al. 2024). A few species, such as *Aculeataaquatica*, *Atrokylindriopsissetulosa*, and *Melanoctonatectonae*, can also be distinguished from other Herpotrichiellaceae species by their conidiogenesis and conidial morphology (Ma et al. 2015; Tian et al. 2016; Dong et al. 2018). Although ITS and LSU sequence analyses support the placement of these three species in Herpotrichiellaceae, reliable genes (e.g., *rpb2*, *tef1*, and *tub2*) may provide better resolution for distinguishing species within this family. Due to the recent increase in the number of Herpotrichiellaceae members and the apparent polyphyletic nature of their characteristics, the delineation of the family remains inconclusive (Tian et al. 2021; Crous et al. 2023). Currently, most sequence data of Herpotrichiellaceae strains in GenBank are available only for ribosomal genes, including ITS, LSU, and SSU, while data for protein-coding genes remain limited. Although the ITS and LSU can be used for linking sexual and asexual morphs and delimitating species, their classification is often unclear or placed in unsupported branches (Untereiner and Naveau 1999; Quan et al. 2020, 2022; Costa et al. 2023; Thitla et al. 2023; Torres-Garcia et al. 2023). Polyphyletic genera (i.e., *Capronia*, *Cladophialophora*, *Exophiala*, *Fonsecaea*, and *Rhinocladiella*) and ambiguous species within the Herpotrichiellaceae family are still waiting for resolution, particularly with the inclusion of newly collected specimens. Likewise, sequences of protein-coding genes (e.g., *act*, *rpb2*, *tef1*, and *tub2*) from additional collections, as well as the re-sequencing of generic types within this family and polyphyletic strains, are required to clarify their phylogenetic affinity and species delineation.

Members of the family Herpotrichiellaceae play various ecological roles, including endophytic, epiphytic, fungicolous, hypersaprobic, lichenicolous, pathogenic, rock-inhibiting, and saprobic taxa, on different substrates, as listed in Table 3. These fungi not only interact with their hosts in various ways but are also considered opportunistic organisms that inhabit natural environments (Crous et al. 2007; Quan et al. 2024). Members of the family Herpotrichiellaceae are known for their biochemical and ecological adaptations that enable survival in extreme environments, often linked to the production of protective compounds such as melanin (de Hoog et al. 2011; Zhan et al. 2011; Coleine and Selbmann 2021; Coelho et al. 2022; Quan et al. 2024; de León et al. 2025). For example, *Cladophialophoraexuberans*, *Exophialadermatitidis*, *E.pisciphila*, and *E.phaeomuriformis* produce melanin, which enables them to thrive under high-radiation, high-temperature or oligotrophic conditions (Badali et al. 2008; de Hoog et al. 2011; Zhan et al. 2011; Cordero and Casadevall 2017; Benmessaoud et al. 2023; da Silva et al. 2023; de León et al. 2025). Although this study employed culture-based techniques to isolate and identify endolichenic fungi within Herpotrichiellaceae, many taxa from this family and others likely remain undetected due to their unculturable nature, low abundance, or specific growth requirements. Future research incorporating metaomics approaches, including metagenomics and metatranscriptomics, could provide a more comprehensive view of fungal diversity, functional gene expression, and ecological interactions within the lichen holobiont. These tools offer promising avenues to uncover hidden fungal lineages and understand their metabolic roles in lichen symbiosis and environmental adaptation.

**Table 3.** Updated genera in Herpotrichiellaceae with their life mode, habitat, and isolation sources.

Genera	Life mode	Habitat	Isolation source	References
*Aciculomyces*	Saprobic	Freshwater	Fluvial sediments	Torres-Garcia et al. (2023)
*Aculeata*	Saprobic	Freshwater	Submerged wood	Dong et al. (2018)
*Atrokylindriopsis*	Saprobic and endolichenic	Terrestrial	Dead branches of an unidentified broadleaf tree and lichen (*Parmotrema* sp.)	Ma et al. (2015); this study
*Capronia*	Epiphytic, fungicolous and hypersaprobic, lichenicolous, and saprobic	Terrestrial	Fungi, lichen, litter, plants, soil, water, and wood	Müller et al. (1987); Etayo (2002); Untereiner (2000); Untereiner and Naveau (1999); Untereiner et al. (2011); Marincowitz et al. (2008); Teixeira et al. (2017); Phookamsak et al. (2019); Sánchez et al. (2019); Roux et al. (2020); Hollinger and Lendemer (2021); Tian et al. (2021)
*Cladophialophora*	Endophytic, fungicolous lichenicolous, pathogenic, rock-inhabiting, and saprobic	Terrestrial and freshwater	Fluvial sediments, lichen, plants, rock, sawdust, soil, wood, warm-blooded animal, and water	Teixeira et al. (2017); Quan et al. (2020); Thitla et al. (2023, 2024); Chang et al. (2023); Cometto et al. (2023)
*Exophiala*	Endophytic, fungicolous, pathogenic, rock-inhabiting, and saprobic	Aquatic, marine, and terrestrial	Cold blooded animals, fungi, litter, plants, rock, sediments, soil, various substrates (i.e., contact lens, plastic foil, soap container, etc.), warm- blooded animals, and water	de Hoog et al. (2011); Teixeira et al. (2017); Quan et al. (2020); Tian et al. (2021); Thitla et al. (2022); Ide-Pérez et al. (2024)
*Fonsecaea*	Epiphytic, pathogenic, and saprobic	Terrestrial	Cold blooded animal, insect, lichen, litter, plants, soil, warm-blooded animal, and wood	Vicente et al. (2012, 2014); de Azevedo et al. (2015); Teixeira et al. (2017); de Souza Lima et al. (2020); Sousa et al. (2024)
*Marinophialophora*	Saprobic	Marine	Associated with *Halocyphina* species on decaying mangrove wood	Li et al. (2018)
*Melanoctona*	Saprobic	Terrestrial	Decaying wood	Tian et al. (2016)
Minimelanolocus*	Saprobic	Aquatic and terrestrial	Litter, plants, and submerged wood	Castañeda Ruíz et al. (2001, 2003); Zhang et al. (2009); Ma et al. (2011); Heredia et al. (2014); Hernández-Restrepo et al. (2013); Tian et al. (2016)
*Petriomyces*	Rock-inhabiting	Terrestrial	Rock	Thitla et al. (2023)
*Phialophora*	Endolichenic, pathogenic, and saprobic	Freshwater and terrestrial	Cold blooded animal, fluvial sediments, food, lichen, plants, soil, warm-blooded animal, water, and wood	Li et al. (2017); Jiang et al. (2017); Song et al. (2021); Tian et al. (2021); Ply et al. (2023); Torres-Garcia et al. (2023); this study
*Phaeoannellomyces*	Pathogenic	Terrestrial	Warm-blooded animal	McGinnis et al. (1985)
Pleomelogramma*	Saprobic	Terrestrial	Decaying woody plants	Spegazzini (1909)
*Rhinocladiella*	Epilithic, epiphytic, lichenicolous, pathogenic, and saprobic	Terrestrial	Honey, insects, plants, soil, warm-blooded animal, and wood	Arzanlou et al. (2007); Hernández-Restrepo et al. (2016); Teixeira et al. (2017); de Souza Lima et al. (2020); Quan et al. (2020); Roeun et al. (2022); Sousa et al. (2024)
*Thysanorea*	Saprobic	Aquatic and terrestrial	Plants and wood	Arzanlou et al. (2007); Tian et al. (2016); Dong et al. (2018); Hernández-Restrepo et al. (2020); Yang et al. (2023)
*Uncispora*	Saprobic	Freshwater and terrestrial	Plants and submerged wood	Sinclair and Morgan-Jones (1979); Li et al. (2014); Liu et al. (2018)
*Valentiella*	Saprobic	Terrestrial	Carton runway galleries built by *Aztecabrevis* ants and plants	Nepel et al. (2014); Bezerra et al. (2022)
*Veronaea*	Endophytic, epiphytic, pathogenic, and saprobic	Freshwater and terrestrial	Animal dung, cold-blooded animal, plants, soil, warm blooded animal, and wood	Ellis (1976); Papendorf (1976); Moustafa and Abdul-Wahid (1990); Arzanlou et al. (2007); Soares and Barreto (2008); Chlebicki (2009); Sang et al. (2011)Soto et al. (2017)Coleman et al. (2018); Chandrasiri et al. (2021); Su et al. (2023); Yang et al. (2023); this study

* Sequence data are not available.

## Supplementary Material

XML Treatment for Atrokylindriopsis racemosospora

XML Treatment for Phialophora chinensis

XML Treatment for Veronaea endolichena

## ﻿Additional information

### Use of AI

No use of AI was reported.

### Author ORCIDs

Chanokned Senwanna https://orcid.org/0000-0002-1008-4514

Jaturong Kumla https://orcid.org/0000-0002-3673-6541

Pratthana Kodchasee https://orcid.org/0000-0003-0432-4910

Nutchanan Duangkon https://orcid.org/0009-0008-5769-0117

Nakarin Suwannarach https://orcid.org/0000-0002-2653-1913

### Data availability

All of the data that support the findings of this study are available in the main text.
